# Temporal Arcuate Relaxing Retinotomy for Persistent Full-Thickness Macular Holes: Anatomical and Functional Assessment

**DOI:** 10.3390/jcm15020863

**Published:** 2026-01-21

**Authors:** Luca Ventre, Erik Mus, Antonio Valastro, Gabriella De Salvo, Michele Reibaldi

**Affiliations:** 1 Department of Ophthalmology, Beauregard Hospital, Azienda USL della Valle d’Aosta, Via L. Vaccari 5, 11100 Aosta, Italy; lventre@ausl.vda.it (L.V.);; 2 Department of Ophthalmology, University of Turin, Via Cherasco 23, 10126 Turin, Italy; 3 Southampton Eye Unit, University Hospital Southampton Foundation Trust, Southampton SO16 6YD, UK; gabrielladesalvo@gmail.com; 4 Department of Clinical and Experimental Sciences, University of Southampton, Southampton SO17 1BJ, UK

**Keywords:** full-thickness macular hole, persistent full-thickness macular hole, relaxing retinotomy, pars plana vitrectomy

## Abstract

**Background:** Evidence guiding secondary repair of persistent full-thickness macular holes (FTMHs) remains limited and heterogeneous. Temporal arcuate relaxing retinotomy has been described as a salvage maneuver intended to increase temporal retinal compliance, yet functional safety data are scarce. We report consecutive real-world outcomes of temporal arcuate relaxing retinotomy for persistent FTMHs after failed standard repair(s). **Methods:** Retrospective consecutive case series of patients with persistent FTMH after ≥1 pars plana vitrectomy (PPV) with internal limiting membrane (ILM) peeling, treated with repeat PPV and temporal arcuate relaxing retinotomy. Outcomes included OCT (Optical Coherence Tomography)-confirmed closure after gas absorption and best-corrected visual acuity (BCVA, logMAR), ellipsoid zone (EZ) status, retinotomy-site morphology on OCT/fundus autofluorescence (FAF), and safety/functional outcomes (systematic scotoma symptom inquiry; Humphrey visual field testing when feasible). Exact binomial 95% confidence intervals (CI) were calculated for proportions. **Results:** Nine eyes (median age 70 years; range 55–76) underwent temporal arcuate relaxing retinotomy for persistent FTMH. Minimum linear diameter ranged 412–1037 µm (median 613 µm). OCT-confirmed closure was achieved in 7/9 eyes (77.8%; 95% CI 40.0–97.2) at a mean follow-up of 5.9 months (range 2–12). BCVA improved in 8/9 eyes (88.9%; 95% CI 51.8–99.7); mean BCVA improved from 1.26 ± 0.51 logMAR pre-operatively to 0.61 ± 0.18 logMAR at last follow-up (mean change −0.64 logMAR; Wilcoxon signed-rank test *p* = 0.011). As a sensitivity analysis, the paired *t*-test yielded *p* = 0.008. Humphrey visual fields were obtained in 6/9 eyes; one patient reported a new paracentral nasal scotoma, which was subjectively well tolerated. **Conclusions:** In this small consecutive series, temporal arcuate relaxing retinotomy was associated with a 78% closure rate and mean BCVA improvement in eyes with persistent FTMH after failed standard repair(s), with limited symptomatic scotoma reporting in those assessed. Given the retrospective design, small cohort, and incomplete standardized functional testing, larger comparative studies with uniform functional endpoints (microperimetry, RNFL/GCL metrics, and systematic perimetry) are needed to define patient selection, reproducibility, and relative performance versus contemporary salvage options.

## 1. Introduction

Pars plana vitrectomy (PPV) combined with internal limiting membrane (ILM) peeling and intraocular gas tamponade remains the standard surgical treatment for full-thickness macular holes (FTMHs), with reported closure rates commonly ranging from approximately 80% to near 100% depending on case mix and hole size [1,2,3,4,5,6]. Despite these favorable outcomes, anatomical failure still occurs, and macular holes may either remain open after the initial attempt or reopen after a period of documented closure [6,7]. FTMHs that do not close after primary surgery are commonly described as persistent (or refractory), whereas those that reopen after at least four weeks of confirmed closure are considered recurrent [6,8].

Secondary repair is challenging for several reasons. Residual ILM may be limited or absent, reducing feasibility of repeat peeling or flap techniques, and reoperation generally carries lower anatomical success than primary surgery [9]. Nevertheless, reintervention can be justified because meaningful anatomical and functional gains remain possible and the natural course of an open hole may involve enlargement with progressive atrophy and visual deterioration [10,11,12,13].

A broad range of surgical strategies has been proposed, including revision PPV with extension of ILM peeling [14], biologic adjuvants such as autologous plasma/platelet products [15], practical secondary-repair approaches [16], subretinal fluid injection [17], fluid–gas exchange [18], retinal massage [19], microdrain techniques [12,20], macular buckling in selected settings [21], and tissue “plug” approaches such as autologous neurosensory retinal free flap (autologous retinal transplant) [22], human amniotic membrane grafting [23], ILM free flap transplantation [24], and lens capsular flap transplantation [25,26]. Evidence remains largely retrospective and heterogeneous, with limited head-to-head comparisons and inconsistent standardized reporting of functional safety outcomes [7,27].

Relaxing retinotomy represents a distinct salvage approach that aims to increase local retinal compliance via a controlled full-thickness incision adjacent to the hole, thereby reducing constraining tangential forces and facilitating edge approximation [6,28,29,30,31,32]. In 2013, Charles et al. described a temporal arcuate full-thickness retinotomy placed adjacent to the macular hole for large holes that remained open after standard surgery [29]. The rationale for performing a temporal arcuate retinotomy is to increase retinal elasticity beyond that achievable with ILM peeling alone, thereby allowing the temporal retinal bridge to relax and shift into a position that promotes macular hole closure [6,29]. The temporal location is intended to align with axonal orientation near the horizontal raphe and potentially reduce risk of papillomacular bundle injury and symptomatic nasal visual field defects [29]. A related but anatomically different approach, nasal parafoveal retinotomy, has also been reported and includes discussion of scotoma symptoms and perimetry findings [30,33].

In this retrospective case series, we aimed to assess both anatomical closure rates and functional outcomes following temporal arcuate relaxing retinotomy for the management of persistent FTMHs. The present study does not propose a new procedure. It provides a consecutive case series describing real-world outcomes, with explicit reporting of postoperative assessment and available functional safety data.

## 2. Material and Methods

This retrospective, single-center interventional case series assessed the effectiveness of temporal arcuate relaxing retinotomy in eyes with persistent full-thickness macular holes (FTMHs). Eligible patients were recruited at Beauregard Hospital (Aosta, Italy) between November 2022 and April 2024.

Informed consent was obtained from all participants for the surgical procedure and for the use of anonymized clinical data for research purposes, as temporal arcuate relaxing retinotomy represents an established salvage option for persistent/refractory full-thickness macular holes; for this retrospective analysis of anonymized data, formal Ethics Committee approval was not required according to local institutional policy, and the study was conducted in accordance with the principles of the Declaration of Helsinki.

The data presented in this study are available from the corresponding author upon reasonable request; the data are not publicly available due to privacy and institutional restrictions.

Inclusion criteria included patients with persistent FTMH after one or more prior PPV with ILM peeling (with or without ILM flap).

Exclusion criteria were (1) clinically significant macular comorbidity likely to confound outcomes (advanced diabetic retinopathy, retinal vein occlusion with macular sequelae, end-stage age-related macular degeneration); (2) inability to comply with postoperative face-down positioning. Because relaxing retinotomy approaches may be associated with postoperative scotomas and potential neuroretinal consequences, advanced glaucoma was considered a relative contraindication in clinical decision-making consistent with previous reports [30].

Baseline data collected included demographics (age, sex), laterality, macular hole etiology, previous repair attempts, and lens status. FTMH size was assessed with spectral-domain OCT (Spectralis; Heidelberg Engineering). Minimum linear diameter (MLD) was defined as the smallest aperture diameter across macular scans, and basal diameter as the maximal width at the level of the retinal pigment epithelium when available. OCT assessment (closure type and ellipsoid zone status) was performed by a single experienced grader using predefined criteria.

BCVA was recorded and converted to logMAR for analysis.

All procedures were performed by a single surgeon (LV) using 23-gauge or 25-gauge transconjunctival PPV (Constellation, Alcon) under 3D visualization (NGENUITY, Alcon). Residual ILM near the intended retinotomy area was stained using viewILM dye (Alchimia, Italy). If residual ILM was present, an extended circumferential peel was performed and documented (yes/no), consistent with revision strategies [14].

**Figure 1:**
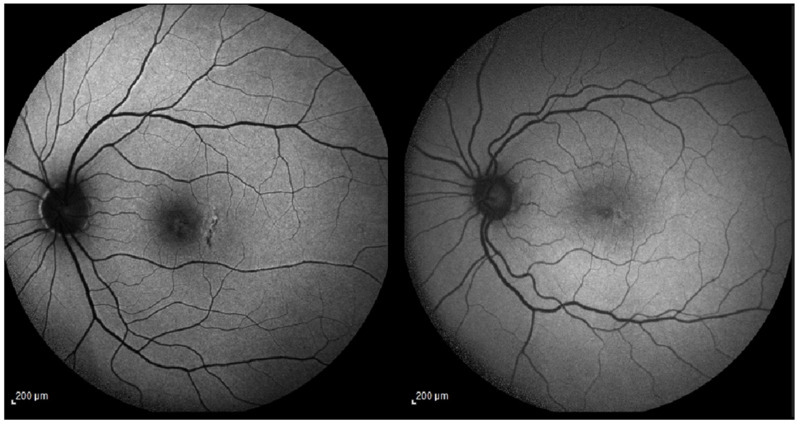
(**Left**) (patient 2): Fundus autofluorescence (AF) shows a well-defined area of retinal pigment epithelium (RPE) alteration temporal to the fovea, corresponding to the arcuate full-thickness relaxing retinotomy (one hole diameter temporal to the macular hole edge). (**Right**) (patient 1): AF demonstrates minimal RPE disturbance at the temporal retinotomy site, with almost imperceptible changes. Abbreviations: AF, autofluorescence; RPE, retinal pigment epithelium.

A full-thickness temporal arcuate (semicircular) relaxing retinotomy was created using vertical or horizontal scissors, positioned approximately one hole diameter temporal to the macular hole edge and centered along the temporal horizontal meridian (Figure 1), consistent with Charles’ technique [29]. Fluid–air exchange and tamponade were then performed using sulphur hexafluoride (SF6) or perfluoropropane (C3F8); silicone oil was used in selected circumstances based on clinical considerations. Patients were instructed to maintain face-down positioning for four days after surgery (unless otherwise clinically indicated).

Postoperative follow-up visits were scheduled at day 1, week 1, and at 1, 3, 6, and 12 months after surgery, and thereafter as clinically indicated. Starting at postoperative month 1, patients were systematically asked at each visit about newly developed scotomas or subjective visual field disturbances, reflecting concerns described in prior retinotomy series. Automated perimetry (Humphrey Field Analyzer 3, HFA3; Carl Zeiss Meditec) was offered after the secondary procedure when feasible with the 30-2 program (SITA Standard), monocularly in the operated eye. Reliability indices (fixation losses, false-positive and false-negative responses) were recorded and considered when interpreting results. Standardized microperimetry and peripapillary RNFL/GCL thickness metrics were not routinely available for all patients in this retrospective series and were not used as endpoints.

The primary outcome was OCT-confirmed anatomical closure of the FTMH after tamponade absorption and at final follow-up. Secondary outcomes included (1) change in BCVA (logMAR) from baseline to last follow-up; (2) EZ integrity in eyes achieving closure; (3) retinotomy-site morphology on OCT and FAF; (4) functional safety outcomes including patient-reported scotoma and HVF findings when available.

Anatomical outcome was graded on OCT as type 1 closure (complete foveal tissue continuity without a neurosensory defect) or type 2 closure (flattened edges with a persistent foveal defect). In this study, ‘closure’ refers to type 1 closure.

Descriptive statistics were used (mean ± SD, median and range). Proportions were reported with exact binomial 95% CI. Pre- vs. postoperative BCVA was primarily compared using the Wilcoxon signed-rank test; *p* < 0.05 was considered statistically significant. Given the sample size, results are presented as hypothesis-generating. Given the small sample size, changes in BCVA were primarily analyzed using the Wilcoxon signed-rank test. A paired *t*-test was additionally computed as a sensitivity analysis.

## 3. Results

Nine consecutive eyes from nine patients were included (seven male, two female), with a median age of 70 years (range 55–76). Etiology was idiopathic in six eyes, traumatic in two eyes (patients 5 and 9), and post-retinal detachment repair in one eye (patient 4). Two traumatic cases were long-standing (≥6 months), including one documented for more than four years (patient 9). All eyes were pseudophakic at baseline.

FTMH MLD ranged from 412 to 1037 µm (median 613 µm). All eyes had previously undergone at least one PPV with ILM peeling (with or without ILM flap) without closure, consistent with persistent/refractory FTMH status [6,7,8,9].

All eyes underwent repeat PPV and temporal arcuate relaxing retinotomy. Residual ILM at the retinotomy site was documented in 4/9 eyes, in which an extended circumferential ILM peel was performed. Tamponade agents included SF6 (4/9), C3F8 (4/9), and silicone oil (1/9).

After surgery, all patients adhered to a face-down positioning regimen for at least 4 days. A summary of baseline characteristics, macular hole features, surgical details, and postoperative outcomes is provided in Table 1.

**Figure 2:**
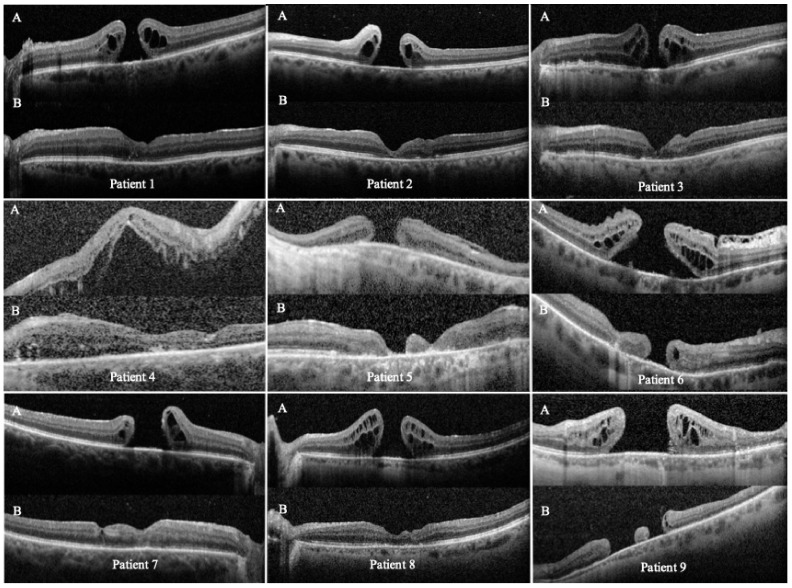
Preoperative (**A**) and postoperative (**B**) OCT scans of the nine eyes that underwent temporal arcuate relaxing retinotomy for persistent full-thickness macular hole (FTMH) repair. Each panel shows cross-sectional scans through the fovea and the temporal arcuate retinotomy site. Abbreviations: OCT, optical coherence tomography; FTMH, full-thickness macular hole.

OCT-confirmed closure was achieved in 7/9 eyes (77.8%; exact 95% CI 40.0–97.2). Mean follow-up was 5.9 months (range 2–12) (Figure 2). Among closed holes, EZ restoration was complete or partial (granular) in 4 eyes. Retinotomy-site imaging (OCT through the retinotomy and FAF when available) did not show signs of pathology at the incision site in the cases with documentation.

**Figure 3:**
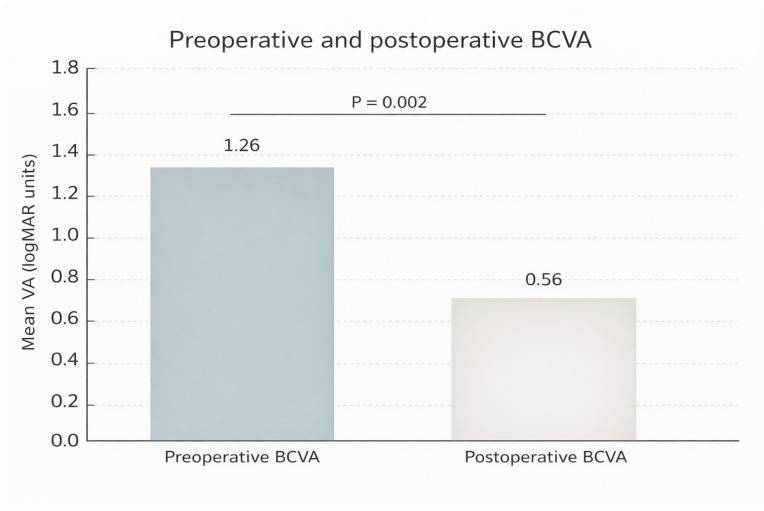
Preoperative and postoperative best-corrected visual acuity (BCVA; mean ± SE, logMAR) in nine eyes undergoing temporal arcuate relaxing retinotomy for repair of persistent full-thickness macular hole (FTMH). Abbreviations: VA, visual acuity; BCVA, best-corrected visual acuity; logMAR, logarithm of the minimum angle of resolution.

BCVA improved in 8/9 eyes (88.9%; exact 95% CI 51.8–99.7). Mean BCVA improved from 1.26 ± 0.51 logMAR at baseline to 0.61 ± 0.18 logMAR at last follow-up (Wilcoxon signed-rank test, *p* = 0.011). As a sensitivity analysis, the paired *t*-test yielded *p* = 0.008 (Figure 3).

One patient reported a new paracentral nasal scotoma that was subjectively well tolerated. This patient experienced better functionality and quality of life with a postoperative paracentral scotoma than with a central scotoma due to FTMH.

**Figure 4:**
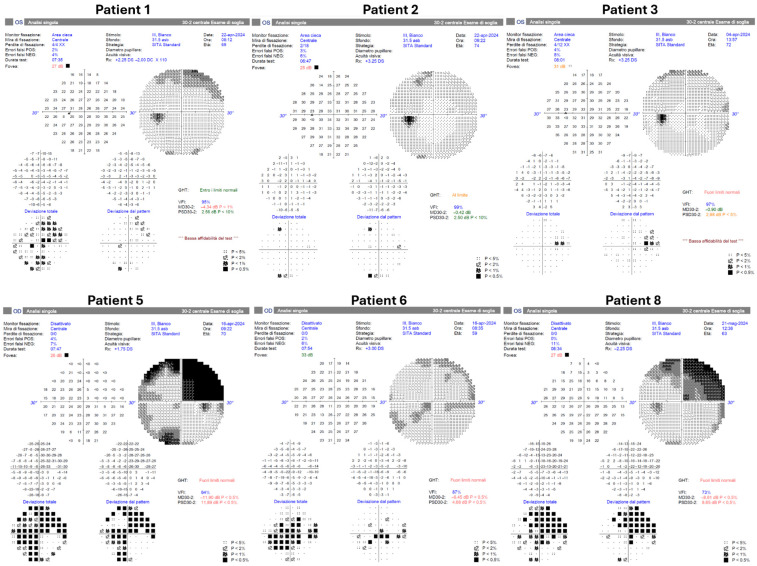
Postoperative Humphrey visual field testing was successfully performed in six patients using the HFA3 perimeter (Carl Zeiss Meditec, Dublin, CA, USA). Patients 4, 7, and 9 did not undergo postoperative visual field examination.

HVF testing was obtained in 6/9 eyes (Figure 4). Four tested eyes showed no scotomas. One tested eye demonstrated a superotemporal scotoma considered consistent with pre-existing chorioretinal atrophy. Three eyes did not undergo HVF testing.

Two eyes did not close at last follow-up: one idiopathic persistent hole with short follow-up of 2 months (patient 6) and one (patient 9) very chronic traumatic hole (documented > 4 years). Additional structural features (including basal diameter and edge configuration) were not consistently available for all cases.

The temporal arcuate relaxing retinotomy successfully enabled closure even in one very large, long-standing FTMH (patient 5, expanded case study ahead).

### Expanded Case Study

Patient 5 was a 70-year-old man with a long-standing traumatic right-eye FTMH following severe blunt trauma two decades before presentation. The FTMH was chronic (lasting more than 1 year), measuring 1037 µm in aperture diameter, with flat borders and inferonasal chorioretinal atrophy. Several years ago, this traumatized eye had already undergone a PPV with ILM peeling, without achieving FTMH closure.

We performed a 23-G pars plana vitrectomy with extended ILM peeling and a temporal relaxing retinotomy to attempt to close this large macular hole, followed by gas tamponade (SF6) and 7 days of face-down positioning.

**Figure 5:**
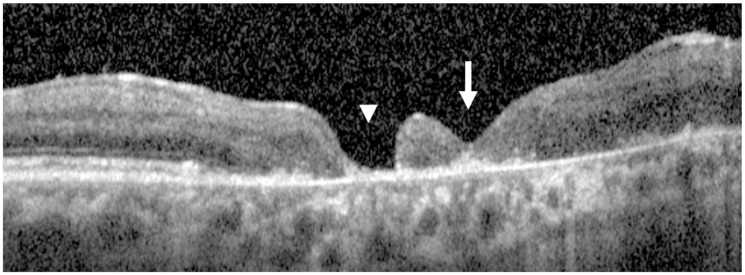
Postoperative OCT scans of patient 5. The white arrow indicates the completely closed macular hole, while the arrowhead marks the persistently open retinotomy site. Despite the open retinotomy and marked inferonasal chorioretinal atrophy, partial retinal slippage resulted in complete macular hole closure.

At follow-up, the FTMH achieved complete anatomic closure, with BCVA improving from counting fingers at 30 cm to 20/100. The retinotomy site remained open, but the most significant finding is that the macular hole completely closed, confirming the partial slippage of the retina to close the hole, considering the notable inferonasal chorioretinal atrophy (Figure 5).

## 4. Discussion

In this small consecutive series of persistent/refractory FTMH after failed PPV/ILM peeling-based repair(s), temporal arcuate relaxing retinotomy combined with repeat PPV achieved OCT-confirmed closure in 78% of eyes and was associated with significant BCVA improvement. Available reports on retinotomy-based salvage for persistent/refractory macular holes are limited and heterogeneous; a concise comparison of the main published retinotomy approaches and reported outcomes is provided in Table 2 [28,29,30,31,32].

A structured symptom inquiry and HVF testing when feasible identified limited symptomatic scotoma reporting in those assessed. These findings align with prior reports suggesting that relaxing retinotomy can facilitate closure in selected difficult cases, while overall evidence remains limited and heterogeneous [6,28,29,30].

The reported outcomes of currently available techniques suggest that reoperation can be justified in recurrent or refractory FTMHs. However, head-to-head comparisons across procedures remain difficult because randomized controlled trials and large prospective comparative studies are scarce [7]. Accordingly, the best operative strategy remains uncertain, and no standardized management pathway for recurrent or refractory full-thickness macular holes has been established [7].

This study does not claim technical novelty. Its contribution is incremental: consecutive real-world outcomes in a defined salvage population and transparent reporting of postoperative assessment and available functional safety findings, addressing a recognized limitation of the current literature on secondary repair strategies [7,27].

Persistent holes after ILM peeling-based repair likely reflect insufficient retinal compliance and ongoing tangential forces that prevent stable edge approximation [6,27]. A controlled full-thickness arcuate incision temporal to the hole is intended to increase local compliance and allow temporal retinal mobility beyond that achievable with ILM peeling alone, thereby facilitating closure [29]. The temporal arcuate design along the horizontal raphe was proposed to reduce the likelihood of severing major axonal trajectories and to limit the risk of symptomatic nasal field defects compared with approaches closer to the papillomacular bundle [29]. While gliotic responses after retinal injury have been proposed as a contributor to closure, mechanistic inference remains speculative without dedicated biomarkers and standardized structure–function correlation [28].

Two eyes did not close. One was a very chronic traumatic hole documented for more than four years. Chronicity and atrophic substrate are recognized challenges in secondary repair and may limit the biomechanical and biological capacity for edge re-approximation [7,10,11,12,27]. The second non-closure case had only two months of follow-up, raising the possibility that the observation window was insufficient to capture delayed closure. Given the small cohort, these findings are descriptive and do not identify predictors; however, they underscore the need for future studies to standardize reporting of chronicity, basal diameter, hole configuration, and timing of closure assessment [9,14].

Relaxing retinotomy is inherently tissue-sacrificing and may be associated with RPE disturbance at the incision site and postoperative scotomas [6,30]. In Tsipursky et al.’s series of nasal parafoveal retinotomy, scotomas were reported and objective perimetry demonstrated defects in a subset, though functional impact was generally outweighed by improvements in central vision [20]. In the present series, symptom inquiry was systematic from postoperative month 2 onward and HVF was performed in two-thirds of eyes; one patient reported a new paracentral scotoma that was well tolerated.

Nonetheless, the lack of uniform microperimetry and RNFL/GCL metrics limits conclusions regarding neuroretinal safety, and larger prospective studies should incorporate standardized functional endpoints [30]. Careful selection is advisable in eyes with significant macular comorbidity or advanced glaucoma, where additional neuroretinal compromise may be clinically consequential [30].

Secondary repair strategies include revision PPV with ILM peeling extension [14], biologic augmentation such as autologous plasma products [15], subretinal fluid injection [17], fluid–gas exchange [18], and tissue plug/graft approaches such as autologous retinal transplant [22], lens capsule flap transplantation [25,26], ILM free flap transplantation [24], and amniotic membrane grafting [23]. Tissue plug techniques may be favored for very large or chronically persistent holes when ILM is exhausted, while compliance-based maneuvers (including relaxing retinotomy) may be considered when the surgeon aims to promote closure without introducing graft material into the defect, preserving the option of subsequent plug strategies if needed [22,25,27]. This series does not compare approaches and does not support superiority claims.

This study is limited by its retrospective design, small cohort, heterogeneous tamponade selection, and variable follow-up intervals. A control group was not available, precluding comparison against other salvage techniques [7,27]. OCT grading was not performed by masked independent graders, and inter-observer variability could not be assessed. Functional testing was not standardized across all eyes; microperimetry and RNFL/GCL thickness were not routinely obtained, limiting safety inference [30]. Quantitative measures of retinotomy size and precise morphology were not systematically recorded and should be incorporated into future studies to improve reproducibility and to explore structure–function correlations.

## 5. Conclusions

In this small consecutive series, temporal arcuate relaxing retinotomy as part of repeat PPV was associated with a 78% closure rate and significant BCVA improvement in persistent FTMHs after failed standard repair(s). Larger comparative studies with standardized imaging-derived retinotomy metrics and uniform functional endpoints (perimetry, microperimetry, RNFL/GCL analyses) are needed to define reproducibility, patient selection, and relative performance versus contemporary salvage options [7,17,22,25,27,30].

## Figures and Tables

**Table 1:** Baseline characteristics, surgical details, and postoperative outcomes of eyes undergoing temporal arcuate relaxing retinotomy for macular hole.

Patient	Gender	Age	Eye	Lens Status	Surgical Procedure	Hole Diameter FTMH (µm)	Preoperative BCVA (LogMAR)	Postoperative BCVA (LogMAR)	Follow-Up (Months)	FTMH Closure	Etiology
1	M	68	LE	Pseudophakic	PPV + enlargement ILMP + TRR + SF6	412	1	0.5	9	Yes	Idiopathic
2	M	73	LE	Pseudophakic	PPV + enlargement + TRR + C3F8	612	1	0.5	5	Yes	Idiopathic
3	M	72	LE	Pseudophakic	PPV + enlargement + TRR + C3F8	457	1	0.4	4	Yes	Idiopathic
4	M	73	LE	Pseudophakic	PPV + enlargement ILMP + TRR + SO	Not available	2.3	0.5	6	Yes	Post-RD repair
5	M	70	RE	Pseudophakic	PPV + enlargement ILMP + TRR + SF6	1037	2	0.7	7	Yes	Traumatic
6	F	58	RE	Pseudophakic	PPV + enlargement + TRR + C3F8	610	1	1	2	No	Idiopathic
7	M	76	RE	Pseudophakic	PPV + enlargement + TRR + SF6	911	1	0.5	12	Yes	Idiopathic
8	F	63	LE	Pseudophakic	PPV + enlargement + TRR + SF6	499	1	0.7	5	Yes	Idiopathic
9	M	55	LE	Pseudophakic	PPV + enlargement ILMP + TRR + C3F8	939	1	0.7	3	No	Traumatic

Abbreviations: M, male; LE, left eye; RE, right eye; C_3_F_8_, perfluoropropane; FTMH, full-thickness macular hole; ILMP, internal limiting membrane peeling; PPV, pars plana vitrectomy; SF_6_, sulfur hexafluoride; SO, silicone oil. TRR, temporal relaxing retinotomy; BCVA, best corrected visual acuity; LogMAR, logarithm of minimum angle of resolution.

**Table 2:** Literature comparison of retinotomy-based salvage approaches for persistent/refractory macular holes.

Study	Design	Retinotomy Type/Location	*n* (Eyes)	Population	Key Adjuncts	Tamponade	Closure (%)	VA Outcome	Functional Safety Reporting	Notes
Reis et al. [28]	Case report/series	Relaxing retinotomy (varied)		Failed standard surgery					NR	Early reports
Charles et al. [29]	Case series	Temporal arcuate full-thickness		Large/persistent MH	ILMP				Limited/NR	Foundational temporal technique
Tsipursky et al. [30]	Case series	Nasal parafoveal relaxing retinotomy		Refractory MH	Suction/drainage maneuver			Reported	HVF subset; scotomas reported	Nasal location
Knight et al. [31]	Case report	Nasal retinotomy		Reopened MH					NR	
Karacorlu et al. [32]	Case report	Double arcuate relaxing retinotomy		Large MH					NR	
Present study	Retrospective consecutive	Temporal arcuate relaxing retinotomy	9	Persistent after ≥1 PPV/ILMP	ILMP extension in 4/9	SF6/C3F8/SO	77.8	Improved; *p* = 0.002	Symptoms systematic; HVF 6/9	Real-world feasibility

## Data Availability

The original contributions presented in this study are included in the article. Further inquiries can be directed to the corresponding author(s). Data from this study were presented in part at the International Edition of XXIII Congress Italian Society of Vitreoretinal Surgery, Trieste, Italy, 8–10 June 2023. Luca Ventre and Erik Mus are co-first authors for this study. All authors have read and agreed to the published version of the manuscript.
